# The effect of histone deacetylase inhibitors on AHSP expression

**DOI:** 10.1371/journal.pone.0189267

**Published:** 2018-02-01

**Authors:** Mohammad Ali Okhovat, Katayoun Ziari, Reza Ranjbaran, Negin Nikouyan

**Affiliations:** 1 Diagnostic Laboratory Sciences and Technology Research Center, School of Paramedical Sciences, Shiraz University of Medical Sciences, Shiraz, Iran; 2 Department of Pathology, Be’sat Hospital, AJA University of Medical Sciences, Tehran, Iran; University of Naples Federico II, ITALY

## Abstract

Alpha-hemoglobin stabilizing protein (AHSP) is a molecular chaperone that can reduce the damage caused by excess free α-globin to erythroid cells in patients with impaired β-globin chain synthesis. We assessed the effect of sodium phenylbutyrate and sodium valproate, two histone deacetylase inhibitors (HDIs) that are being studied for the treatment of hemoglobinopathies, on the expression of AHSP, BCL11A (all isoforms), γ-globin genes (HBG1/2), and some related transcription factors including GATA1, NFE2, EKLF, KLF4, and STAT3. For this purpose, the K562 cell line was cultured for 2, 4, and 6 days in the presence and absence of sodium phenylbutyrate and sodium valproate. Relative real-time qRT-PCR analysis of mRNA levels was performed to determine the effects of the two compounds on gene expression. Expression of all target mRNAs increased significantly (*p* < 0.05), except for the expression of BCL11A, which was down-regulated (*p* < 0.05) in the cells treated with both compounds relative to the levels measured for untreated cells. The findings indicated that sodium valproate had a more considerable effect than sodium phenylbutyrate (*p* < 0.0005) on BCL11A repression and the up-regulation of other studied genes. γ-Globin and AHSP gene expression continuously increased during the culture period in the treated cells, with the highest gene expression observed for 1 mM sodium valproate after 6 days. Both compounds repressed the expression of BCL11A (-XL, -L, -S) and up-regulated GATA1, NFE2, EKLF, KLF4, STAT3, AHSP, and γ-globin genes expression. Moreover, sodium valproate showed a stronger effect on repressing BCL11A and escalating the expression of other target genes. The findings of this in vitro experiment could be considered in selecting drugs for clinical use in patients with β-hemoglobinopathies.

## Introduction

Alpha-hemoglobin stabilizing protein (AHSP) is an erythroid-specific protein that acts as a molecular chaperone for α-globin chains and forms a stable but reversible complex with the free alpha chains of hemoglobin, stabilizing them to prevent precipitation in cells [1].

AHSP-alpha globin interactions could be a potential β-thalassemia modifier and lead to diversity in hematological and clinical symptoms of patients. For instance, some individuals with β-thalassemia trait are asymptomatic and show insignificant symptoms, possibly as a result of excess free α-globin chains stabilization by AHSP. Moreover, simultaneous mutations that affect AHSP function or expression in β-thalassemia could result in more disease severity [2, 3].

It has been found that an increase in the α-globin to β-globin chain synthesis ratio leads to AHSP overexpression. Consequently, AHSP can bind to more free alpha chains, decreasing the severity of clinical symptoms caused by sedimentation of excess unbound α-globin chains [4].

The clinical signs and symptoms of β-thalassemia could be impressively improved even by a partial increase in the non-α to α globin chain ratio. Several drugs such as 5-azacytidine, hydroxycarbamide, erythropoietin, butyrate derivatives, and hemin have been examined both in vitro and in vivo to reactivate γ-chain synthesis [5]. Among these drugs, only hydroxycarbamide has been approved for the treatment of sickle cell disease (SCD), and since its approval, the clinical manifestation of SCD patients has improved noticeably [6]. However, the effect of these new therapies on β-thalassemia is limited. This phenomenon may be related to the higher level of HbF required in β-thalassemia to yield clinical results comparable to those observed for sickle cell anemia [7].

The γ-globin genes (HBG1 and HBG2) are normally expressed in the fetal liver, spleen, and bone marrow. Two γ-globin chains together with two α-globin chains constitute fetal hemoglobin (HbF), which is normally replaced by adult hemoglobin (HbA) in the year following birth. It has been shown that butyrate compounds and other short-chain fatty acids (SCFAs) enhance the formation of hemoglobin F. SCFAs such as arginine butyrate, sodium phenylbutyrate, and sodium isobutyramide act as inhibitors of histone deacetylases (HDACs) and lead to changes in chromatin structure and reprogram gene expression [8–12]. Previous studies have shown that these compounds result in the up-regulation of important erythroid transcription factors such as GATA binding factor 1 (GATA1), nuclear factor erythroid 2 (NFE2), and Kruppel like factor 4 (KLF4). Overexpression of GATA1, NFE2, and KLF4 genes has been reported to cause up-regulation of γ-globin gene [13–16].

The discovery of the quantitative trait locus B-cell lymphoma-leukemia A (BCL11A) on chromosome 2p16 recognized this factor as a significant regulator of γ-globin gene expression. Subsequent studies have shown that BCL11A is a powerful modulator of human HbF levels and its down-regulation in adult erythroid cells results in a vigorous induction of γ-globin gene expression [17–19]. Erythroid Kruppel like factor (EKLF/KLF1) has been shown to repress γ-globin gene expression and decrease human gamma-globin to beta-globin expression ratio thorough direct upregulation of BCL11A in human and mouse adult erythroid progenitors [20, 21].

In different experiments, a direct relationship has been observed between AHSP gene and transcription factors such as GATA1, NFE2, and EKLF [22–25]. Additionally, Cao et al. revealed that AHSP gene is a target of signal transducer and activator of transcription 3 (STAT3) and that its expression is directly modulated by STAT3 signaling. They detected multiple STAT3 binding sites in the AHSP promoter upon α-globin overloading in K562 cells [26].

Valproic acid is a short-chain fatty acid (SCFA) that has histone deacetylases inhibitory activity [27] and is currently used for the treatment of seizure and bipolar disorders. Recently, valproic acid has been shown to inhibit HDACs in therapeutic doses. Clinical observations have indicated that HbF increases in epileptic patients treated with valproic acid. Valproic acid has also been used in some patients with SCD to increase HbF [13, 28–30].

The γ-globin gene is weakly expressed in adults, and even its up-regulation by inducing drugs cannot completely compensate for the lack of β-globin synthesis and neutralize excess free α-globin chains. Given the significant role of AHSP in neutralizing the destructive effect of excess free alpha chains in cells and in improving the clinical status of patients with impaired β-globin genes, it seems essential to identify pharmaceutical agents that can simultaneously increase both AHSP and γ-globin mRNA and contribute to the overexpression of these genes at therapeutic doses. Therefore, in this study, we assessed the effect of sodium phenylbutyrate and valproic acid sodium salt on the expression of AHSP, BCL11A (-XL, -L, -S), γ-globin genes (HBG1/2), and some related transcription factors including GATA1, NFE2, EKLF, KLF4, and STAT3.

## Materials and methods

### Cell culture and induction

Cells of the human erythroleukemia K562 (CCL-243) cell line were obtained from ATCC (Manassas, VA, USA). The cells were cultured in RPMI-1640 Glutamax medium (Gibco, Life Technologies, USA) supplemented with 10% heat-inactivated fetal bovine serum (Gibco, Life Technologies, USA) with the addition of penicillin-streptomycin in a 5% CO_2_ humidified atmosphere at 37 °C.

For experiments, the cells were seeded at a density of 3 × 10^5^ cells/4 ml culture media in 6-well cell culture plates (Corning^®^ Costar^®^, Sigma, Germany) and cultured for 2, 4, and 6 days in the presence or absence of different concentrations of sodium phenylbutyrate (NB) (C_10_H_11_NaO_2_) (Abcam, UK), valproic acid sodium salt (NV) (C_8_H_15_NaO_2_) (Abcam, UK), and hemin (Sigma-Aldrich, Germany). The following concentrations of inducers were used: 0.5 and 1 mM sodium phenylbutyrate, 0.5, 1, and 2 mM valproic acid sodium salt, and 50 μM hemin. Each compound was tested in triplicate, and the whole experiment was repeated 5 times in different weeks. The results of treatments were statistically compared with the control levels.

### Benzidine staining

Untreated, NB-treated, NV-treated and hemin-treated K562 cells were washed by PBS 1X and then stained in a benzidine solution including 0.6% (w/v) benzidine, 2% (v/v) H_2_O_2_, and 12% (v/v) acetic acid. At least 1000 cells were counted at each time point after treatment using a light microscope to assess the percent of cells that appeared blue owing to the presence of heme-containing globin tetramers.

### RNA isolation, DNase treatment and cDNA synthesis

After incubation, the cells were harvested by centrifugation and the growth medium was removed. Total RNA from cells was isolated using TRIzol Reagent (Life Technologies, USA) according to the manufacturer’s instructions. Isolated RNA was dissolved in sterile diethylpyrocarbonate (DEPC)-treated double-distilled water and quantified in a NanoDrop spectrophotometer by absorption at 260 nm and 280 nm. Then, genomic DNA was removed from the isolated RNA in a 10 μl reaction containing 2 μg total RNA, 2 U DNase I, RNase-free (Thermo Scientific, USA) and 1X DNase I reaction buffer with MgCl_2_.

Complementary DNA (cDNA) was synthesized using the PrimeScript RT Reagent Kit (Perfect Real Time) (TaKaRa, Japan). RNA was reverse transcribed into cDNA in a 10 μl reaction containing 1 μg DNase I-treated total RNA, 1X PrimeScript buffer, 0.5 μl PrimeScript RT Enzyme Mix I (40 U RT Enzyme), 25 pmol oligo(dT) primers and 200 pmol random hexamers.

### Relative real-time qRT-PCR

Real-time qRT-PCR reactions were performed in QIAGEN's real-time PCR cycler (Rotor-Gene Q) (QIAGEN, Germany) with SYBR Green PCR Master Mix (SYBR Premix Ex Taq^™^ II, Tli RNaseH Plus) (TaKaRa, Japan). The real-time qRT-PCR reaction (total volume of 20 μl) contained 0.3 μM forward and reverse primers, 1X SYBR Premix Ex Taq II, and 1 μl (100 ng) cDNA. The amplification profile involved initial denaturation at 95 °C /30 seconds and 40 cycles of denaturation at 95 °C/5 seconds, annealing at 57 °C/30 seconds, and elongation at 72 °C/30 seconds, followed by a melting curve stage. The real-time qRT-PCR reaction was performed for all genes of interest as targets and β-actin (ACTB) as the endogenous reference gene for each sample. Table 1 shows the list of target genes and sequences of the primer pairs for each gene. Each set of reactions included a negative control minus RT Enzyme and another without cDNA (NTC). All reactions, including the no-template control (NTC), were conducted in triplicate. Melting curve analysis verified the real-time qRT-PCR amplification, and electrophoresis on 2% agarose gel validated it as well.

**Table 1:** 

Gene	Accession Number	Primer Pair	Product Length (bp)	Product Melt Temperature (C°)
**ACTB**	NM_001101.3	Forward: CCTCCATCGTCCACCGCAA	197	83.10
		Reverse: GCTGTCACCTTCACCGTTCCA		
**GATA1**	NM_002049.3	Forward: CTTCATCACTCCCTGTCC	104	84.20
		Reverse: AGAGGAATAGGCTGCTGA		
**AHSP**	NM_016633.3 NM_001318221.1 NM_001318222.1	Forward: CCGCAGGATTGAAGGAGTT	163	85.35
		Reverse: GCCTTGTCTCGCTCTTGG		
**HBG 1** **HBG 2**	NM_000559.2 NM_000184.2	Forward: GGCAAGGTGAATGTGGAAG	111	85.35
		Reverse: AGAGGCAGAGGACAGGTT		
**BCL11A-XL** **BCL11A-L** **BCL11A-S**	NM_022893.3 NM_018014.3 NM_138559.1	Forward: GTCTCGCCGCAAGCAAGG	100	85.70
		Reverse: GCCGTGGTCTGGTTCATCATCT		
**NFE2**	NM_006163.2 NM_001136023.2 NM_001261461.1	Forward: CGATGCTGAATCTCTTGA	129	86.65
		Reverse: AAGGTATAGTTGGAGTGG		
**EKLF/ KLF1**	NM_006563.3	Forward: CCAAATAAACGGACTCAG	139	87.40
		Reverse: TAATATCAGCCACAATAAGG		
**KLF4-Isoform 1** **KLF4-Isoform 2**	NM_001314052.1 NM_004235.5	Forward: GGACGGCTGTGGATGGAA	135	88.25
		Reverse: ATGTGTAAGGCGAGGTGGT		
**STAT3- Isoform 1** **STAT3- Isoform 2** **STAT3- Isoform 3**	NM_139276.2 NM_003150.3 NM_213662.1	Forward: AGTTCTCCTCCACCACCAA	364	86.90
		Reverse: GTCTTACCGCTGATGTCCTTC		

Target genes and sequences of the oligonucleotide primer pairs used for real-time qRT-PCR amplification.

The amplification efficiency of each gene was determined using dilution series of the gene from pooled cDNA of the control samples. Each dilution series was amplified in triplicate, and the obtained C_T_ values were used to construct the standard curve. Plots were composed of the log of the template concentration versus the C_T_, and the amplification efficiency (E) was calculated from the slope of the line using the following equation: E = 10(-1/S)– 1 (S = slope of the standard curve). The amplification efficiency of all target genes (AHSP, HBG1/2, BCL11A (-XL, -L, -S), GATA1, NFE2, EKLF, KLF4, and STAT3) and β-actin (ACTB) as the endogenous reference gene were determined. All expression levels were evaluated using the relative quantification method described by Pfaffl and coworkers [31].

### Western blotting analysis

Whole protein was extracted from K562 cells using cell lysis Radioimmunoprecipitation assay buffer (RIPA buffer including 150 mM NaCl, 1.0% NP-40, 0.5% sodium deoxycholate, 0.1% SDS (sodium dodecyl sulphate), 50 mM Tris-HCl, pH 8.0, Protease inhibitors). For BCL11A, nuclear protein was extracted by Nuclear Extraction Kit (Abcam, UK). Approximately 30 to 50 μg of protein from samples was denatured and separated by 10% or 15% sodium dodecyl sulfate polyacrylamide gel electrophoresis (SDS-PAGE) and transferred to Polyvinylidene difluoride (PVDF) membranes (Bio-Rad, USA). After blocking by 5% BSA in TBST buffer (Tris-buffered saline, 0.1% Tween 20), immunoblotting was performed with rabbit antibodies against STAT3, AHSP, BCL11A (-XL, -L, -S), HBG globin chains, and β-actin (Abcam, UK). Horseradish peroxidase (HRP) conjugated secondary antibodies and Enhanced Chemiluminescence (ECL) kit (Amersham Biosciences, UK) were used for detection and visualization of protein levels.

### Statistical analysis

A relative expression analysis of the target genes, a group-wise comparison, and a statistical analysis of relative expression results were performed using pairwise fixed reallocation randomization tests with REST 2009 software (Relative Expression Software Tool). SPSS 20.0 (SPSS Inc., Chicago, IL) was used to analyze the data. An independent-sample t-test was performed to compare the groups statistically. Data are presented as the mean plus standard error range, and *p* values < 0.05 were considered statistically significant.

## Results

Maturation and erythroid differentiation of K562 cells were identified by benzidine staining. When K562 cells differentiate towards erythroid cells, increased positive cell percentage of benzidine staining were detected. We used hemin as the control of maturation and differentiation. Only 4% of untreated K562 cells stained with benzidine after 6 days. The addition of hemin led to the increased appearance of benzidine-positive cells (54%±2.16) as result of erythroid differentiation. However, the percent of benzidine-positive cells was significantly more in sodium valproate-treated cells (86%±3.87) and sodium phenylbutyrate-treated cells (78%±3.12) compared to hemin-induced cells (*p* = 0.001).

The relative expression ratio (R) was presented as the fold change in gene expression, normalized to an endogenous reference gene compared with the control. Therefore, a value of R > 1.0 was considered to represent overexpression of mRNA in the treated cells (Sample) compared with the untreated cells (Control), whereas R <1.0 represented down-expression.

**Fig 1:**
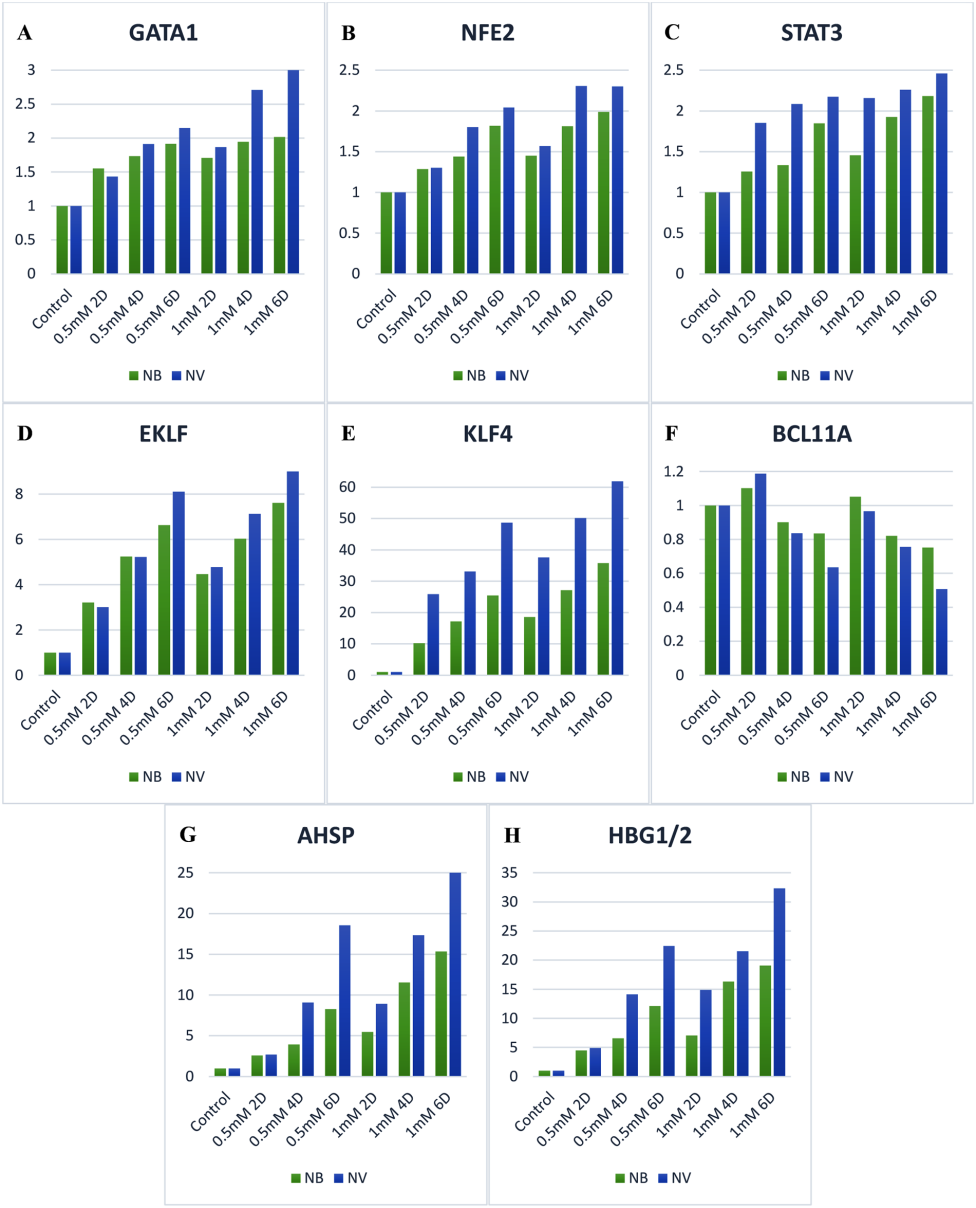
The effect of sodium phenylbutyrate and sodium valproate on the expression of AHSP, BCL11A, γ-globin genes (HBG 1/2) and erythroid transcription factors. Both compounds led to significant induction of AHSP, γ-globin genes (HBG1/2) and erythroid transcription factors in K562 cells (*p* < 0.05). However, BCL11A was significantly repressed due to treatment with them (*p* < 0.05). Higher concentrations of the studied HDIs and longer treatment times led to significantly greater repression of BCL11A and higher expression of other target genes (*p* < 0.0005). NB, sodium phenylbutyrate; NV, sodium valproate; 2D, two days of treatment; 4D, four days of treatment; 6D, six days of treatment.

Generally, all target genes in the cells treated with 0.5 mM sodium phenylbutyrate were overexpressed compared with the untreated cells except for BCL11A. After two days of treatment with 0.5 mM sodium phenylbutyrate, γ-globin mRNA was up-regulated by a mean factor of 4.482 (S. E.: 4.374–4.610) (*p* < 0.0005) and AHSP mRNA was overexpressed by a mean factor of 2.578 (S. E.: 2.549–2.603) (*p* < 0.0005) compared with the control group (untreated cells) (Fig 1G and 1H). Other targets, including GATA1, NFE2, EKLF, KLF4, and STAT3, also showed overexpression (*p* < 0.0005) (Fig 1A–1E). The expression ratio for γ-globin and AHSP mRNA increased considerably to 12.126 (S. E.: 11.711–12.444) (*p* < 0.05) and 8.278 (S.E.: 8.056–8.515) (*p* < 0.0005), respectively, after 6 days of incubation. Additionally, other targets showed a significant increase in expression after this period.

In contrast, BCL11A mRNA was down-regulated by a mean factor of 0.835 (S. E.: 0.779–0.857) (*p* = 0.037) after 6 days of incubation with 0.5 mM sodium phenylbutyrate (Fig 1F).

The higher concentration of sodium phenylbutyrate (1 mM) resulted in greater repression of BCL11A and higher expression of other target genes, and this escalation was statistically significant compared with observed at 0.5 mM sodium phenylbutyrate over the same periods of treatment (*p* < 0.0005). After 6 days of treatment with 1 mM sodium phenylbutyrate, γ-globin and AHSP mRNA were up-regulated by mean factors of 19.048 (S. E.: 18.436–19.602) (*p* < 0.0005) and 15.341 (S. E.: 14.929–15.780) (*p* < 0.0005), respectively, compared with the control group. EKLF and KLF4 mRNA were overexpressed by mean factors of 7.615 (S. E.: 7.266–7.989) (*p* < 0.0005) and 35.741 (S. E.: 35.218–36.296) (*p* < 0.0005), respectively. The relative expression ratios of GATA1, NFE2, and STAT3 were 2.015 (S. E.: 1.946–2.065) (*p* < 0.0005), 1.987 (S. E.: 1.898–2.063) (*p* < 0.0005), and 2.183 (S. E.: 2.104–2.269) (*p* = 0.015), respectively.

However, the expression of BCL11A was down-regulated to 0.752 (S. E.: 0.704–0.787) (*p* = 0.001) due to the higher concentration of sodium phenylbutyrate.

Treatment with 0.5 mM valproic acid sodium salt (sodium valproate) contributed to up-regulation of all targets apart from BCL11A. γ-globin and AHSP were overexpressed by mean factors of 4.901 (S. E.: 4.671–5.246) (*p* < 0.0005) and 2.687 (S. E.: 2.603–2.770) (*p* < 0.0005), respectively, after two days of incubation (Fig 1G and 1H). Other targets including EKLF, KLF4, GATA-1, NFE2, and STAT3 also underwent up-regulation (Fig 1A–1E). A longer incubation time in the presence of sodium valproate (0.5 mM) led to more down-regulation of BCL11A and a noticeably higher expression of other targets’ mRNA. The expression ratios of γ-globin and AHSP rose substantially to 22.436 (S. E.: 21.655–23.334) (*p* < 0.0005) and 18.558 (S. E.: 17.877–19.160) (*p* < 0.0005), respectively, after 6 days of treatment. Similarly, EKLF, KLF4, GATA-1, NFE2, and STAT3 were more overexpressed due to an increase in treatment time.

However, BCL11A mRNA was significantly repressed and down-regulated by a mean factor of 0.636 (S. E.: 0.598–0.658) (*p* = 0.005) after six days of incubation with 0.5 mM sodium valproate.

The relative expression ratios of all targets except BCL11A were considerably scaled up owing to the increase in the concentration of sodium valproate to 1 mM, and the differences were statistically significant over the same periods of treatment (*p* < 0.0005). After 6 days of treatment with 1 mM sodium valproate, γ-globin and AHSP mRNA were overexpressed by mean factors of 32.347 (S. E.: 31.005–27.474) (*p* < 0.0005) and 25.611 (S. E.: 24.695–27.474) (*p* < 0.0005), respectively, compared with the untreated cells. The relative expression ratios of EKLF and KLF4 were 9.043 (S. E.: 8.686–9.428) (*p* < 0.0005) and 61.876 (S. E.: 61.401–62.334) (*p* < 0.0005), respectively. GATA1, NFE2, and STAT3 were up-regulated by mean factors of 3.071 (S. E.: 2.804–3.170) (*p* < 0.0005), 2.301 (S. E.: 2.169–2.458) (*p* < 0.0005), and 2.461 (S. E.: 2.204–2.811) (*p* < 0.0005), respectively.

In contrast, the expression of BCL11A was more strongly repressed and down-regulated to 0.507 (S. E.: 0.486–0.524) (*p* < 0.0005) due to the higher concentration of sodium valproate.

The higher concentration of sodium valproate was toxic to K562 cells. The cell viability was measured using Trypan Blue as a vital dye. After two days of treatment with 2 mM sodium valproate, only 60% of the cells were alive. The cell viability decreased significantly to approximately 25% after 4 days of incubation, and finally, all the cells died after 6 days.

After 6 days, treatment with hemin 50 μM contributed to up-regulation of both γ-globin and AHSP gene by mean factors of 4.963 (*p* < 0.05) and 5.852 (*p* < 0.05), respectively. However, in the same period of time, the effect of valproic acid sodium salt 1 mM (R = 32.347 and 25.611) and sodium phenylbutyrate 1 mM (R = 19.048 and 15.341) were significantly stronger in overexpressing these genes compared to hemin (*p* < 0.0005). Moreover, the effect of hemin was weaker in repressing BCL11A (R = 0.921) and overexpressing KLF4 (R = 12.312), GATA-1 (R = 1.350), NFE2 (R = 1.192), and STAT3 (R = 1.410) compared with valproic acid sodium salt (NV) (*p* < 0.05) and sodium phenylbutyrate (NB) (*p* < 0.05). Conversely to the NV and NB, hemin addition resulted in EKLF down-regulation (R = 0.897).

**Fig 2:**
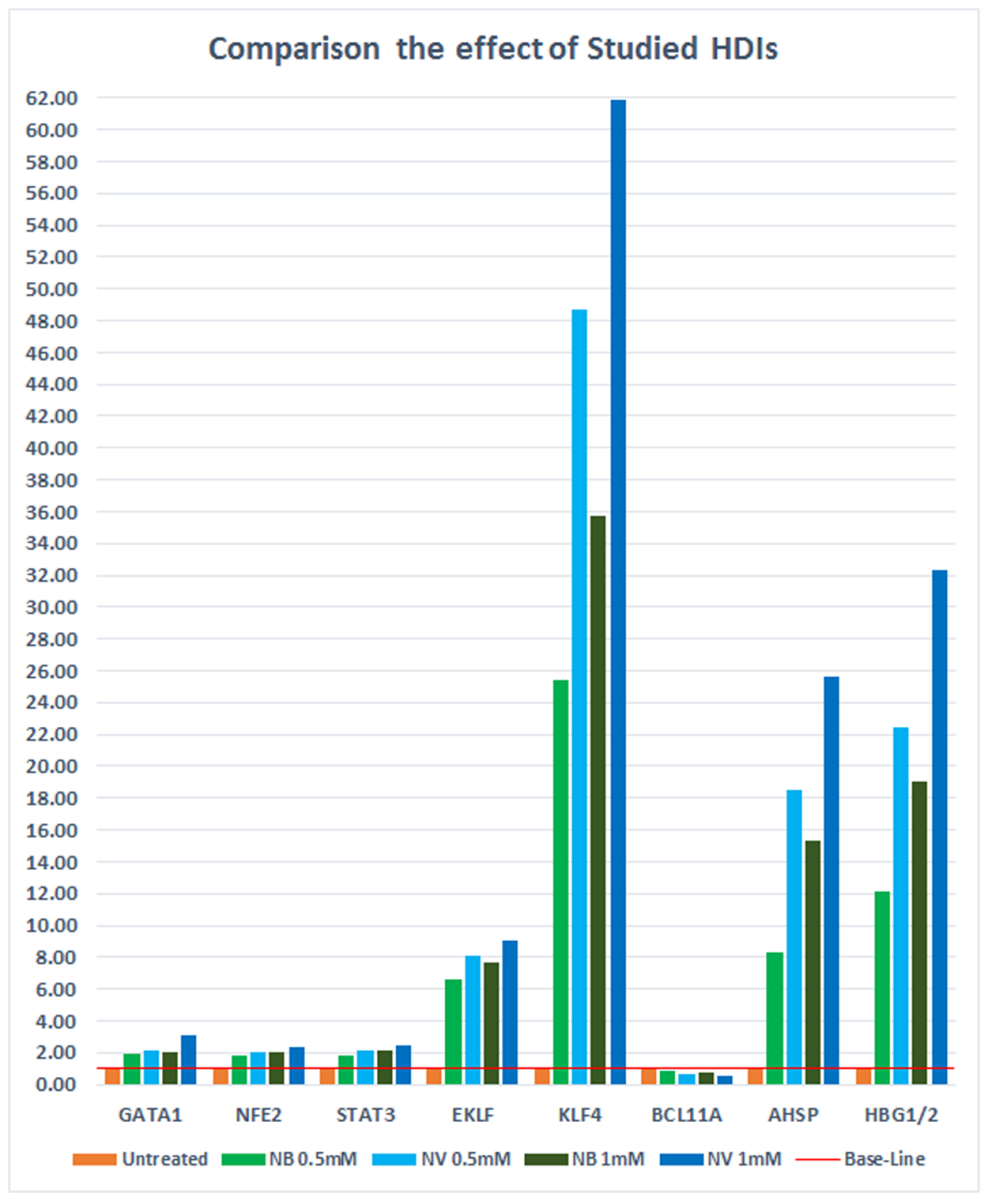
Comparison of the effect of sodium phenylbutyrate and sodium valproate on the expression of AHSP, BCL11A, γ-globin genes (HBG 1/2) and erythroid transcription factors after six days of treatment. Sodium valproate was significantly more efficient on the down-regulation of BCL11A and the up-regulation of AHSP, γ-globin genes (HBG 1/2), and erythroid transcription factors compared with sodium phenylbutyrate (*p* < 0.0005). NB, sodium phenylbutyrate; NV, sodium valproate.

Generally, the effect of valproic acid sodium salt was significantly stronger in down-regulating BCL11A and up-regulating AHSP, γ-globin, EKLF, KLF4, GATA-1, NFE2, and STAT3 compared with sodium phenylbutyrate, especially at higher concentrations and longer treatment times (*p* < 0.0005) (Fig 2 and S1 File).

**Fig 3:**
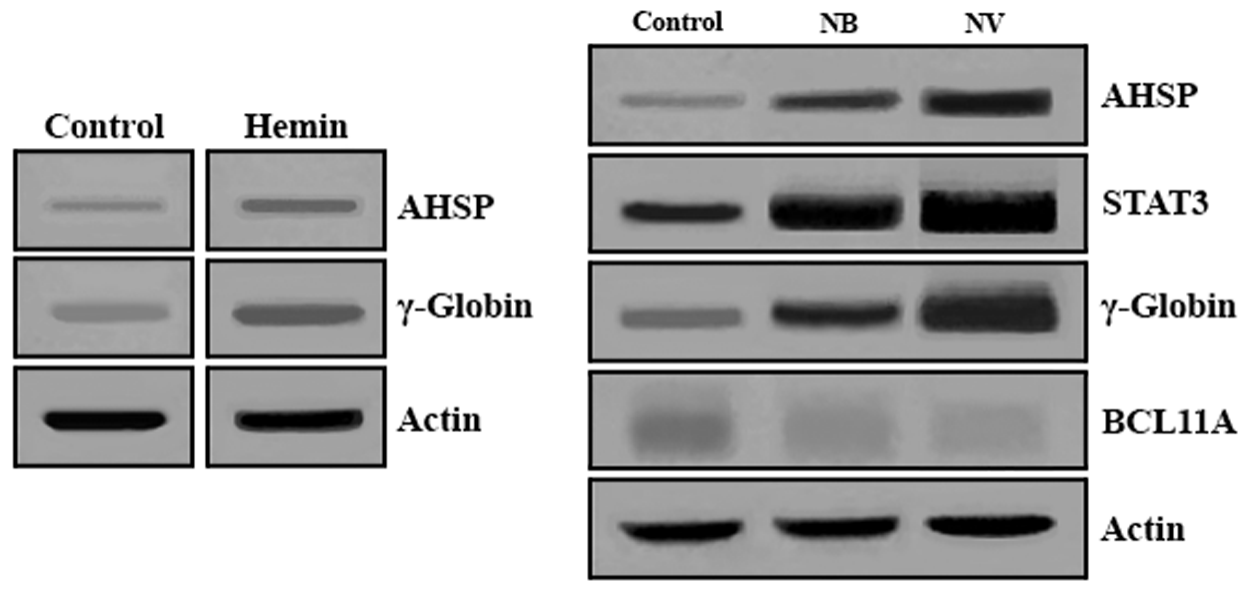
Protein expression analysis by western blotting. K562 cells were cultured with or without sodium phenylbutyrate 1 mM (NB), sodium valproate 1 mM (NV) and hemin 50 μM. After 6 days, the cells were examined for STAT3, AHSP, γ-globin and BCL11A protein expression by Western blot analysis. To normalize protein loading, the blot was hybridized with β-actin antibody.

Western blotting analysis showed that with sodium phenylbutyrate (1 mM) and sodium valproate (1 mM) induction, there were increased amounts of HBG globin chains and conversely decreased amounts of BCL11A protein in the nuclei of K562 cells after 6 days. Similarly, AHSP and STAT3 protein increased significantly in K562 cells treated 6 days with sodium phenylbutyrate and sodium valproate compared to untreated cells. Fig 3 shows that the effect of sodium valproate (1 mM) was considerably more than that of sodium phenylbutyrate (1 mM). Western blotting analysis also revealed the increased amounts of HBG globin chains and AHSP protein after 6 days as a result of hemin (50 μM) induction. However, the effect of sodium valproate (1 mM) and sodium phenylbutyrate (1 mM) were noticeably stronger than that of hemin (50 μM) (Fig 3).

## Discussion

The discovery of AHSP has delivered new perceptions into its role as a chaperone-like molecule specialized for erythroid series which is involved in the formation of functional hemoglobin. Previous studies have shown that AHSP stabilizes newly formed α-globin monomers before binding to β-globin and makes them soluble enough to prevent ineffective erythropoiesis by forming a steady but reversible complex with unbound α-globin chains [1, 32–34].

In 2012, Lim and his colleagues observed that AHSP expression was associated positively with α-globin, β-globin, and excess α-globin expression but it had negative correlations with MCH level and HbF percentage. Their results showed that AHSP upregulates to compensate for the extra free α-globin chains in HbE/β-thalassemia patients [4].

To date, several scientists have investigated the correlation between the AHSP genotype and the severity of β-thalassemia, assuming that AHSP could be a potential β-thalassemia modifier. A significant correlation has been found between the expression of AHSP gene and reduction in the severity of clinical and hematological symptoms in β-thalassemia patients [3, 35–37].

Considering the essential role of AHSP in the phenotype and clinical symptoms of β-thalassemia, this protein has recently been viewed as a new potential therapy for β-thalassemia by many researchers. Therefore, in this study, we investigated the effect of sodium phenylbutyrate and sodium valproate on the kinetic expression of AHSP, BCL11A, γ-globin genes (HBG1/2), and some related transcription factors including GATA1, NFE2, EKLF, KLF4, and STAT3 in the human erythroleukemia cell line K562.

We generally found a significant (*p* < 0.05) increase in the expression of transcription factors and consequent induction of AHSP and γ-globin (HBG1/2) gene expression in the erythroid cell line K562 as a result of treatment with 0.5 mM and 1 mM sodium phenylbutyrate within 2, 4, and 6 days. Conversely, the expression of BCL11A was significantly down-regulated due to this treatment (*p* < 0.05).

Consistent with the study conducted by Kalra et al. [16], we found that butyrate derivatives considerably enhanced the induction and expression of KLF4 and γ-globin gene in the erythroid cell line K562. The overexpression of γ-globin gene is also consistent with the results reported by Chenais et al. [14], who examined the effect of butyric acid. Similarly, Ronzoni et al. showed that histone deacetylase inhibitors (HDIs) such as sodium butyrate could induce the expression of γ-globin genes (HBG1/2) [38].

Moreover, we found that sodium phenylbutyrate resulted in the repression and down-regulation of BCL11A, which in turn contributed to the up-regulation of γ-globin gene expression. Similarly, Chen et al. showed that butyrate induction of γ-globin production in K562 is associated with reduced BCL11A [39].

Generally, the results showed that increasing the expression of erythroid transcription factors and BCL11A repression through treatment with sodium phenylbutyrate could up-regulate the expression of AHSP and γ-globin.

On average, the strongest induction effect on transcription factors was related to KLF4, followed by EKLF, GATA1, STAT3, and NFE2. Similarly, Chenais et al. [14] showed that butyric acid results in the overexpression of GATA1 and NFE2.

We observed that the induction of γ-globin gene expression was significantly higher than that of AHSP in the presence of sodium phenylbutyrate in all groups. This finding may indicate the direct up-regulation of γ-globin through BCL11A repression and the greater impact of transcription factors on γ-globin induction compared with AHSP.

In this study, increasing the concentration of sodium phenylbutyrate led to significantly (*p* < 0.0005) stronger repression of BCL11A and higher induction of other target genes. In addition, a longer incubation time enhanced the effectiveness of sodium phenylbutyrate (*p* < 0.0005). These results correspond to those reported by Chenais et al. [14] concerning the effect of butyric acid on the K562 cell line.

The results suggest that similarly to sodium phenylbutyrate, 0.5 mM and 1 mM sodium valproate significantly repressed BCL11A expression (*p* < 0.05) and enhanced the expression of transcription factors (*p* < 0.05) within 2, 4, and 6 days and accordingly induced the expression of AHSP and γ-globin genes (HBG1/2). In accord with Witt et al. [30], 0.5 mM and 1 mM of sodium valproate up-regulated the expression of γ-globin.

The results indicate that sodium valproate down-regulated the expression of BCL11A and amplified the expression of transcription factors as well as sodium phenylbutyrate, thereby inducing the expression of AHSP and γ-globin. Likewise, the greatest impact on the overexpression of transcription factors was related to KLF4, followed by EKLF, GATA1, STAT3, and NFE2.

In treatment with sodium valproate, as with sodium phenylbutyrate, the up-regulation of γ-globin was considerably higher than that of AHSP, which further substantiates the direct up-regulation of γ-globin through BCL11A repression and the greater impact of transcription factors on the induction of γ-globin compared with the AHSP gene.

The results obtained for all groups indicate a significantly (*p* < 0.0005) stronger effect for sodium valproate compared with sodium phenylbutyrate on the repression of BCL11A and the induction of transcription factors (GATA1, NFE2, EKLF, KLF4, STAT3), γ-globin, and AHSP.

We used hemin as the control of maturation and differentiation. The addition of hemin led to the increased appearance of benzidine-positive cells as result of erythroid differentiation. However, after 6 days, the percent of benzidine-positive cells was significantly more in NV-treated cells and NB-treated cells compared to hemin-induced cells (*p* = 0.001). Moreover, the effect of valproic acid sodium salt (NV) 1 mM and sodium phenylbutyrate (NB) 1 mM were significantly stronger in overexpressing γ-globin (4 to 6 times stronger) and AHSP (3 to 4 times stronger) gene compared to hemin (*p* < 0.0005). Similarly, the effect of hemin was weaker in repressing BCL11A and overexpressing KLF4, GATA-1, NFE2, and STAT3 in comparison with NV and NB (*p* < 0.05).

Western blot analysis revealed the induction effect of sodium phenylbutyrate and sodium valproate on γ-globin expression which was associated with decrease of BCL11A protein in K562 cells. Similarly, sodium phenylbutyrate and sodium valproate increased the amounts of STAT3 and AHSP protein in these cells. Protein expression analysis indicates that sodium valproate have a stronger effect on the induction of γ-globin and AHSP levels in comparison with sodium phenylbutyrate. Western blotting analysis also revealed the stronger effect of sodium valproate and sodium phenylbutyrate on the upregulation of γ-globin and AHSP levels compared with hemin.

GATA1 and NFE2 are erythroid-specific activators that bind to the β-globin locus. Kim et al. showed that GATA1 and NFE2 were required for the expression of the human fetal γ-globin gene [40].

Zhou et al. demonstrated that knockdown of EKLF (KLF1) in human and mouse adult erythroid progenitors results in a marked reduction in BCL11A levels and a decrease in γ- to β-globin gene switching, thereby increasing the amount of γ-globin [20]. In the present study, hemin addition resulted in EKLF down-regulation (0.897) and consequently led to BCL11A repression (0.921). Conversely to the hemin, sodium phenylbutyrate and sodium valproate addition were observed to scale up the expression of EKLF (KLF1) transcription factor; however, this increase did not lead to BCL11A overexpression and γ-globin repression. The up-regulation of EKLF and γ-globin gene is consistent with the results described by Jalali Far et al. [41], who studied the effect of sodium butyrate. One possible reason for this phenomenon is the overexpression of KLF4. Kalra et al. showed that sodium butyrate contributes to γ-globin up-regulation in K562 cells by increasing the expression of KLF4 [16]. Similarly, we found that sodium phenylbutyrate and sodium valproate led to a considerable induction of KLF4, and the fold induction was approximately 5 to 7 times greater than that of EKLF. Moreover, we observed that sodium phenylbutyrate and sodium valproate repressed BCL11A, directly resulting in γ-globin up-regulation. The overexpression of γ-globin and BCL11A repression are consistent with the findings reported by Chen et al. [39], who examined the effect of sodium butyrate on K562 cells and showed that sodium butyrate can induce γ-globin expression through downregulation of BCL11A. Accordingly, these findings suggest that sodium phenylbutyrate and sodium valproate may directly repress BCL11A transcription, enhance the degradation of its mRNA, and/or reduce its translation. However, many unanswered questions regarding the mechanisms of action of these histone deacetylase inhibitors (HDIs) upon EKLF, KLF4, BCL11A and γ-globin expression remain.

In a study conducted in 2005, the AHSP gene was identified as a target of the critical erythroid transcription factor GATA1 and was highly and rapidly induced by it [22]. Additionally, it has been found that AHSP is a direct target of EKLF [24, 42]. Keys et al. proposed that EKLF and GATA1 collaborate as co-regulators to control the expression of the AHSP gene during erythropoiesis [43]. Moreover, studies have shown that EKLF is activated by GATA1 [44, 45], which provides an additional secondary mechanism for GATA1 activation of AHSP through EKLF. Guo-wei et al. observed that AHSP expression is highly dependent on NFE2, suggesting that it may play an important role in AHSP gene regulation [23]. Recently, Cao C. et al. showed that AHSP is a target of STAT3 transcription factor and that its induction leads to the overexpression of AHSP [26].

Finally, our findings demonstrated that therapeutic doses of sodium phenylbutyrate and sodium valproate could cause induction and overexpression of γ-globin and AHSP simultaneously in the erythroid cell line K562. These histone deacetylase inhibitors (HDIs) up-regulate the expression of the AHSP gene possibly through the overexpression of GATA1, NFE2, EKLF, and STAT3 transcription factors. Likewise, they enhance the induction of γ-globin synthesis probably by repression of BCL11A and up-regulation of GATA1, NFE2, and KLF4 transcription factors. The overexpression of these two genes reduces clinical and hematological symptoms owing to sedimentation of excess free α-globin chains. In addition, increased HbF production can improve the problems associated with the lack of β-globin, reducing the need for blood transfusion in β-thalassemia patients. Therefore, this production suggests a new route for treating patients with β-thalassemia.

## Supporting information

S1 File Graphs excel file. This file include the individual data points that underlie the results presented in graphs.
(XLSX)
